# Large-Area Metal–Semiconductor Heterojunctions Realized via MXene-Induced Two-Dimensional Surface Polarization

**DOI:** 10.1021/acsnano.2c12684

**Published:** 2023-04-20

**Authors:** Tianchao Guo, Xiangming Xu, Chen Liu, Yizhou Wang, Yongjiu Lei, Bin Fang, Lin Shi, Hang Liu, Mrinal K. Hota, Hala A. Al-Jawhari, Xixiang Zhang, Husam N. Alshareef

**Affiliations:** ∥ Materials Science and Engineering, Physical Science and Engineering Division, King Abdullah University of Science and Technology (KAUST), Thuwal 23955-6900, Saudi Arabia; ‡ Applied Physics, Physical Science and Engineering Division, King Abdullah University of Science and Technology (KAUST), Thuwal 23955-6900, Saudi Arabia; § Department of Physics, King Abdulaziz University, Jeddah 21551 Saudi Arabia

**Keywords:** Ti_3_C_2_T_*x*_ MXene, MoS_2_, thin film, surface polarization, transistor

## Abstract

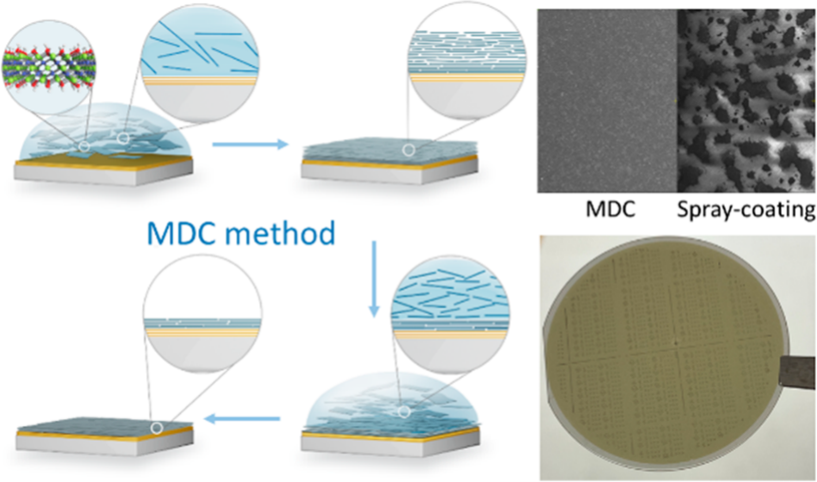

Direct MXene deposition on large-area 2D semiconductor surfaces can provide design versatility for the fabrication of MXene-based electronic devices (MXetronics). However, it is challenging to deposit highly uniform wafer-scale hydrophilic MXene films (e.g., Ti_3_C_2_T_*x*_) on hydrophobic 2D semiconductor channel materials (e.g., MoS_2_). Here, we demonstrate a modified drop-casting (MDC) process for the deposition of MXene on MoS_2_ without any pretreatment, which typically degrades the quality of either MXene or MoS_2_. Different from the traditional drop-casting method, which usually forms rough and thick films at the micrometer scale, our MDC method can form an ultrathin Ti_3_C_2_T_*x*_ film (ca. 10 nm) based on a MXene-introduced MoS_2_ surface polarization phenomenon. In addition, our MDC process does not require any pretreatment, unlike MXene spray-coating that usually requires a hydrophilic pretreatment of the substrate surface before deposition. This process offers a significant advantage for Ti_3_C_2_T_*x*_ film deposition on UV-ozone- or O_2_-plasma-sensitive surfaces. Using the MDC process, we fabricated wafer-scale *n*-type Ti_3_C_2_T_*x*_–MoS_2_ van der Waals heterojunction transistors, achieving an average effective electron mobility of ∼40 cm^2^·V^–1^·s^–1^, on/off current ratios exceeding 10^4^, and subthreshold swings of under 200 mV·dec^–1^. The proposed MDC process can considerably enhance the applications of MXenes, especially the design of MXene/semiconductor nanoelectronics.

MXenes, i.e., two-dimensional (2D) transition metal carbides and nitrides, have been attracting considerable attention since their discovery in 2011.^1^ They offer structural diversity and unique properties,^2−4^ making them attractive in energy conversion,^5^ energy storage,^6^ electronics,^7,8^ sensing,^9^ and electromagnetic interference shielding.^10^ For electronic applications, Ti_3_C_2_T_*x*_ has received significant interest compared to other MXene compositions.^11^ The combination of MXenes and 2D semiconductors such as MoS_2_ facilitates the generation of devices. However, the difficulty of depositing hydrophilic MXene films on hydrophobic semiconductor films (e.g., MoS_2_) hinders its applications in 2D electronics.

Since MXenes are primarily synthesized through chemical etching, followed by molecular delamination in an aqueous solution, MXene-based microdevices are usually prepared using solution deposition methods.^7,11−17^ Because of the Coulomb repulsion among negatively charged MXene flakes, they can easily form a uniform and stable dispersion in an aqueous suspension without using surfactants.^18^ This feature can provide high-quality MXene thin films using a solution process. To date, multiple methods have been developed to prepare MXene films, including drop-casting,^19^ spin-coating,^20,21^ spray-coating,^7,22^ and dip-coating.^23,24^ Among them, drop-casting is a relatively simple, inexpensive deposition method in which the film is formed by transferring the suspension drop onto a substrate, followed by drying.^19,25^ However, this process can only produce thick films (∼μm thickness), limiting its applications in nanoelectronics.^19^ To prepare ultrathin MXene films, spin-coating, spray-coating, and dip-coating have been performed. In a typical spin-coating process, a liquid-based suspension is first spread over the substrate.^26,27^ Most liquid flies off and then evaporates during subsequent high-speed rotation, thus leaving a homogeneous thin film on the substrate.^23,26^ However, the MXene film thickness demonstrates radial variations when deposited over a large substrate by spin-coating.^28,29^ Spray-coating allows the fast fabrication of large-area thin films.^25,26^ In this process, the MXene dispersion is loaded in a spray gun and ejected as aerosol droplets using a high-pressure carrier gas. The velocity and diameter of droplets are controlled using the gas pressure. Once ejected and deposited on a hot substrate, the droplets merge and undergo solvent evaporation, thus aligning dried flakes parallel to the substrate. The percolation network is formed by the movement of the spray gun in the *xy* direction, which results in a continuous film.^30^ Moreover, template masking can be combined for direct patterning as thin films.^31,32^ Although spray-coating is a facile, inexpensive, and rapid method, the percolation issue limits film uniformity and quality.^33^ Moreover, dip-coating has been developed to prepare ultrathin films.^34^ Typically, dip-coating can be divided into four steps: dipping, soaking, deposition and drainage, and solvent evaporation.^24^ In dip-coating, a substrate is soaked in a dispersion for a time period and then lifted out at a constant speed. When the substrate is extracted from the suspension, a thin film is deposited on the substrate owing to the affinity between the substrate and aqueous solution. Note that additional evaporation increases the adhesion between the film and the substrate.^34^ However, in all three ultrathin MXene film processing methods, the substrate must be pretreated to develop a hydrophilic surface (see Supplementary Note). This requirement considerably limits MXene application scenarios, as MXene films may need to be deposited on hydrophobic and UV-ozone (UVO)- or O_2_-plasma-sensitive surfaces (e.g., 2D MoS_2_).^35,36^ Although the addition of proper surfactants in a MXene aqueous solution may facilitate the thin film deposition process on these types of substrates, it is not preferred in high-performance nanoelectronics because of the deteriorated film quality.^26,37^

To address these challenges, we developed a modified drop-casting (MDC) method that eliminated the requirement for surface pretreatment before MXene deposition. Using the MDC method, uniform and continuous ultrathin MXene films with a thickness of 10 nm were deposited on the pristine MoS_2_ surface over a large area without UVO or O_2_ plasma treatment. Formation of the uniform large-area ultrathin MXene film was enabled using the surface polarization of MoS_2_ introduced by rich negative charges on the MXene surface. Combining the MDC method with the lift-off lithography process, we fabricated Ti_3_C_2_T_*x*_–MoS_2_ van der Waals (vdW) heterojunction transistors with an average effective mobility (μ) of ∼40 cm^2^·V^–1^·s^–1^, on/off current ratios (*I*_on_/*I*_off_) exceeding 10^4^, and subthreshold swings (*SS*) of under 200 mV·dec^–1^. The excellent device performance resulted from the flat and intimate vdW interface with MoS_2_, which is directly formed using an aqueous solution process. Therefore, we believe that our MDC method can provide high-quality MXene–MoS_2_ vdW heterojunction interfaces, demonstrating great application potential in 2D electronics.

## Results and Discussion

**Figure 1:**
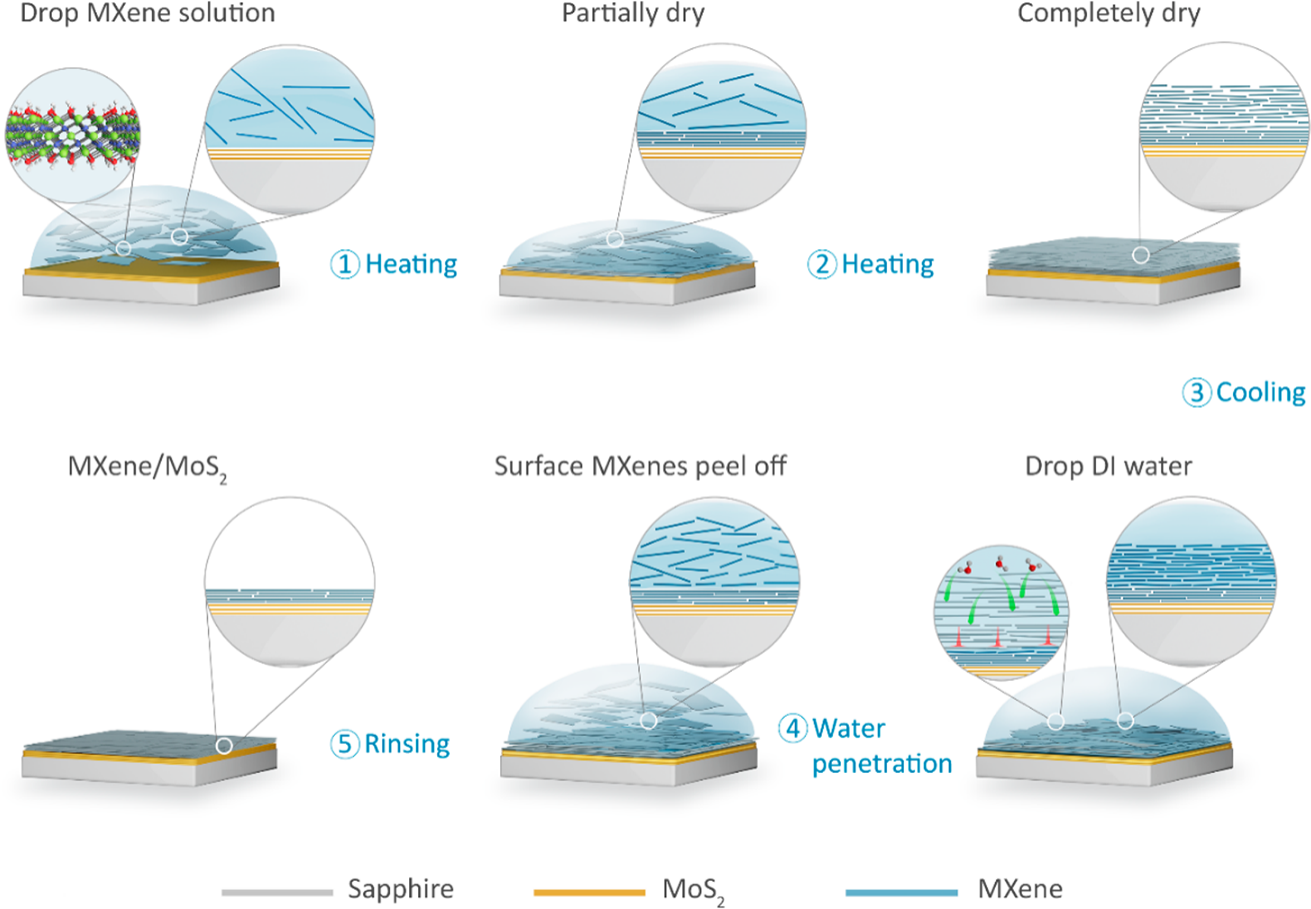
Schematic showing hydrophilic Ti_3_C_2_T_*x*_ thin film deposition on a hydrophobic MoS_2_ surface using our modified drop-casting (MDC) method.

Figure 1 shows the MDC process of Ti_3_C_2_T_*x*_ thin film deposition on the hydrophobic MoS_2_ surface. The 2D atomic layered structure of Ti_3_C_2_T_*x*_ is shown in the inset of Figure 1, where two carbon layers (blue) are located between three Ti layers (green) and two outer layers are chemically bonded surface groups (red and white, representing −F, −OH, and =O). The O and F atoms in terminal groups exhibiting large electronegativity make MXene surfaces highly negatively charged, which may polarize materials on contact with the MXene surface.^38^ The atomic force microscopy (AFM) image of the individual MXene flake’s thickness distribution and the zeta potential distribution of MXene solution are shown in Figures S1 and S2, respectively. Before starting the MDC process, no pretreatment was performed on the MoS_2_ surface to avoid any degradation in MoS_2_ crystal quality. First, the as-synthesized aqueous Ti_3_C_2_T_*x*_ solution was dropped on the MoS_2_ film surface and then dried on a hot plate for a certain time period (Figure 1, steps 1 and 2). During the drying process, as the water evaporated, Ti_3_C_2_T_*x*_ flakes started to laterally attach to the surface of MoS_2_. Once all the water evaporated, a drop-casted thick MXene film was naturally formed on the MoS_2_ surface. Moreover, the Ti_3_C_2_T_*x*_ film was dried on a hotplate for 10 min. After the sample was naturally cooled down to room temperature (Figure 1, step 3), we observed multiple cracks and bulges on the as-drop-casted thick Ti_3_C_2_T_*x*_ film surface using an optical microscope (Figure S3), which can be attributed to the strain effect during the drying process. We used different heating temperatures (70 °C, 90 °C, and 110 °C) and observed similar phenomena (Figure S3). However, the drying temperature had other effects on the Ti_3_C_2_T_*x*_ thin film, which were characterized using Raman spectroscopy (Figure S4). The Raman peaks at ∼205, 375, 610, and 720 cm^–1^ corresponded to Ti_3_C_2_T_*x*_ (Figure S4a), while the peak at ∼153 cm^–1^ in MXene-MDC-110 °C was attributed to the oxidation of the film, in accordance with the results shown in Figure S4b and a previous study.^39^ While the considerably high solvent evaporation rate at 110 °C caused many defects on the film surface, which affected the uniformity and quality of the film (Figure S3), the relatively low solvent evaporation rate at 70 °C severely prolonged the processing time. Therefore, 90 °C was selected as the appropriate temperature in terms of film quality and processing efficiency. X-ray photoelectron spectroscopy (XPS) characterization was further performed to assess the level of MXene oxidation, as shown in Figure S5. A Lorentzian–Gaussian fitting was conducted on the XPS spectrum, revealing a high-resolution Ti 2p spectrum that can be accurately represented by three doublets. Specifically, the Ti 2p_2/3_ peaks were observed centered at 454.9, 455.7, and 457.0 eV, corresponding to Ti–C, Ti(II), and Ti(III), respectively. In addition, the small peak located at 459.0 eV was identified as TiO_2_, which is a normal feature in pristine Ti_3_C_2_T_*x*_.^40^ After complete drying, a thick film was formed. Cross-sectional scanning transmission electron microscopy (STEM) observations (Figure S6) demonstrated the presence of a double-layer structure in the as-drop-casted film, including an upper thick sparse layer and a lower thin dense layer. Then, we dropped deionized (DI) water on the thick Ti_3_C_2_T_*x*_ film for a certain time period to let water molecules penetrate the upper thick sparse layer through surface cracks or bulges (Figure 1, step 4). After rinsing using DI water, we observed that the upper thick Ti_3_C_2_T_*x*_ film was removed (Figure 1, step 5). However, the lower thin layer uniformly remained on the MoS_2_ surface because Ti_3_C_2_T_*x*_ introduced surface polarization (discussed in the next section), which avoided film removal upon repeated water rinsing. Hence, a uniform and continuous Ti_3_C_2_T_*x*_ thin film was obtained on the MoS_2_/sapphire substrate.

**Figure 2:**
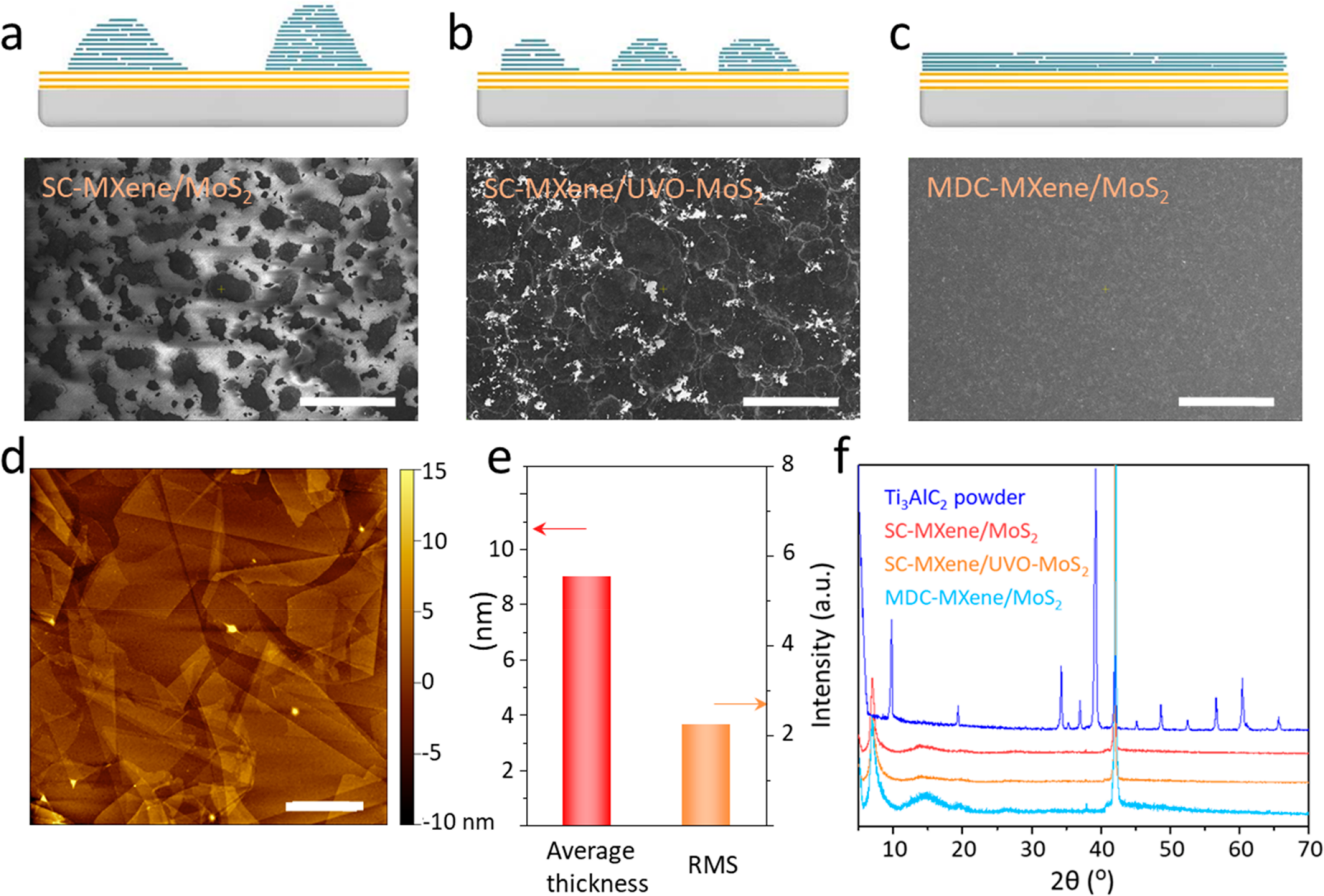
Comparison of Ti_3_C_2_T_*x*_ MXene thin films prepared using different methods. (a) Schematic (top) and SEM image (bottom) of the Ti_3_C_2_T_*x*_ thin film on the pristine MoS_2_ surface deposited using spray-coating (SC-MXene/MoS_2_). (b) Schematic (top) and SEM image (bottom) of the Ti_3_C_2_T_*x*_ thin film on the UVO-treated MoS_2_ surface obtained using spray-coating (SC-MXene/UVO-MoS_2_). (c) Schematic (top) and SEM image (bottom) of the Ti_3_C_2_T_*x*_ thin film on the pristine MoS_2_ surface obtained using the MDC method (MDC-MXene/MoS_2_). (d) AFM images of MDC-MXene/MoS_2_. (e) Average thickness and RMS values of MDC-MXene/MoS_2_. (f) XRD curves of Ti_3_AlC_2_ MAX powder, SC-MXene/MoS_2_ (on sapphire), SC-MXene/UVO-MoS_2_ (on sapphire), and MDC-MXene/MoS_2_ (on sapphire). (In the schematic, blue shows Ti_3_C_2_T_*x*_; yellow shows MoS_2_; dark gray shows the sapphire substrate.) (Scale bars: a–c, 100 μm; d, 2 μm.)

The Ti_3_C_2_T_*x*_ thin film formed on the MoS_2_ surface using the MDC method demonstrates considerably higher quality compared with that deposited by the widely used spray-coating (SC) method, as shown in Figure 2. In the very beginning, we attempted to directly spray-coat the Ti_3_C_2_T_*x*_ MXene thin film on the pristine MoS_2_ surface (without any hydrophilic treatment). However, Ti_3_C_2_T_*x*_ flakes were stacked as isolated multilayer islands rather than continuous films, as demonstrated in the schematic and the corresponding scanning electron microscopy (SEM) image in Figures 2a and S7a–c. The white areas or points in SEM images (Figures 2a,b and S7a–f) because of considerable charge accumulation indicate the absence of conductive Ti_3_C_2_T_*x*_ flakes. Figure S8a,b shows related optical images where the yellow islands belong to Ti_3_C_2_T_*x*_ and the dark gray parts represent the MoS_2_ substrate. The reason for failing to spray-coat a continuous MXene film directly on the MoS_2_ surface might be that the pristine MoS_2_ surface is relatively hydrophobic, with a contact angle of 76.6° (Figure S9a). To facilitate the stacking of Ti_3_C_2_T_*x*_ flakes on the MoS_2_ surface, we performed UVO treatment on the MoS_2_ surface to make it more hydrophilic (the contact angle was reduced to 15°, as shown in Figure S9d), followed by the same SC process. We observed improved Ti_3_C_2_T_*x*_ coverage on the MoS_2_ surface; however, many areas still lacked Ti_3_C_2_T_*x*_ flakes (Figures 2b, S7d–f, and S8c,d). UVO treatment can also oxidize MoS_2_ and degrade its crystal quality, as shown in Figure S10, which must be avoided in high-performance device fabrication.^35^ However, using our MDC method, a uniform Ti_3_C_2_T_*x*_ thin film with full surface coverage was achieved on the pristine MoS_2_ surface, as shown in SEM (Figures 2c and S7g–i) and optical images (Figure S8e,f). Furthermore, the AFM images of these samples, shown in Figures 2d and S11a–c, reported that the MDC-MXene film on MoS_2_ had an extremely low roughness and good continuity. From Figures 2d and S11d, the average thickness and root-mean-square (RMS) value of MDC-MXene/MoS_2_ were found to be 9.0 and 2.25 nm, respectively. Figure 2f shows the X-ray diffraction (XRD) patterns of Ti_3_AlC_2_ MAX phase. powder and the three above-mentioned films. The dominant peak at ∼7° was assigned to the (002) plane of Ti_3_C_2_T_*x*_, and no obvious peak shift was observed for films deposited using other deposition methods.

**Figure 3:**
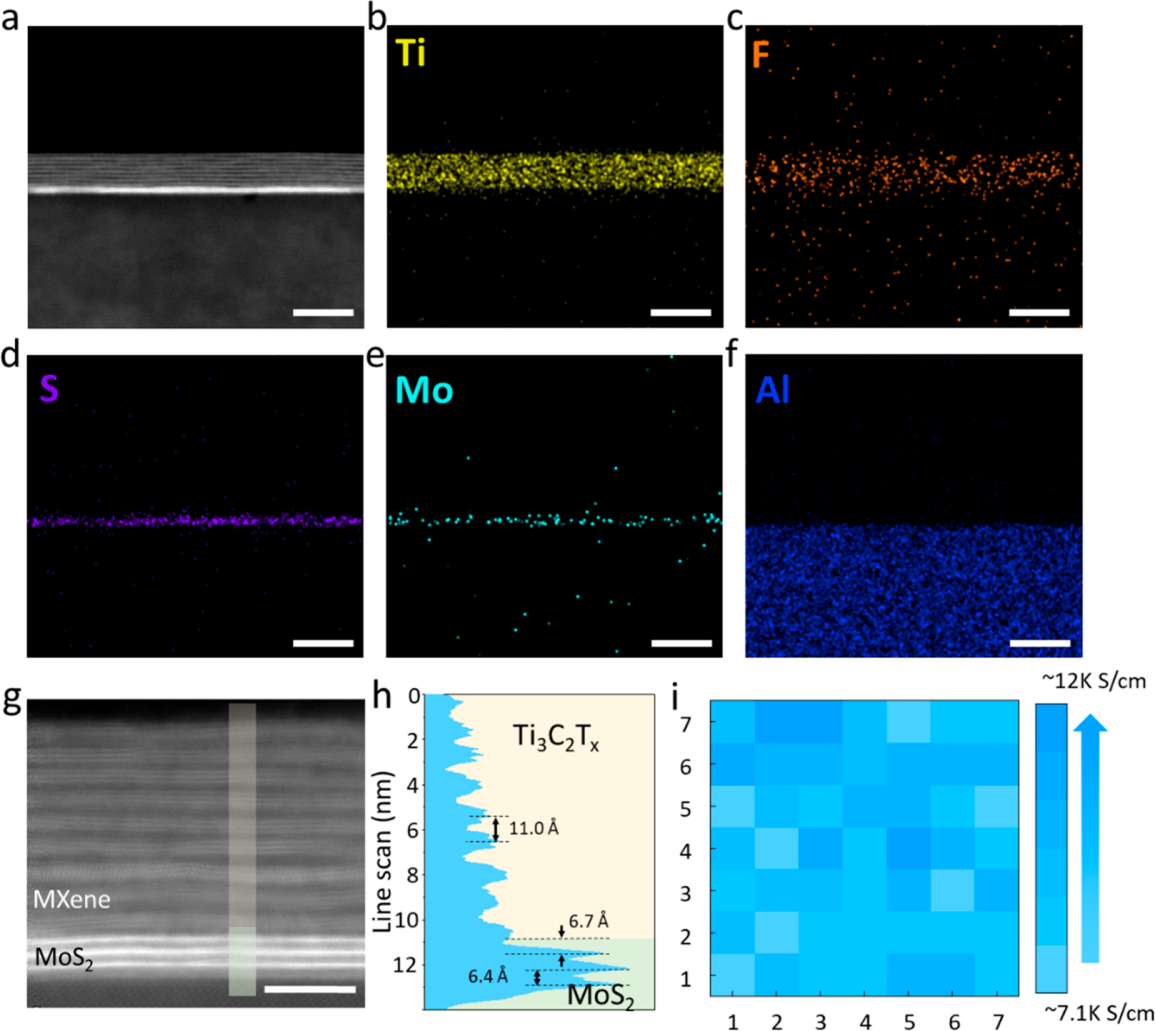
Characterization of the Ti_3_C_2_T_*x*_ thin film deposited on the MoS_2_ surface using the MDC method. (a) Low-magnification cross-sectional STEM image of Ti_3_C_2_T_*x*_/MoS_2_/sapphire. (b) Ti, (c) F, (d) S, (e) Mo, and (f) Al atom EDS mapping. (g) High-resolution cross-sectional STEM image of Ti_3_C_2_T_*x*_/MoS_2_/sapphire. (h) Intensity profile obtained from the line scan in panel g. (i) Mapping of the conductivity of the Ti_3_C_2_T_*x*_ thin film on the MoS_2_ surface. (Scale bars: a–f, 20 nm; g, 5 nm.)

To examine the interface of the Ti_3_C_2_T_*x*_–MoS_2_ vdW heterojunction, we performed a high-resolution cross-sectional dark-field STEM with energy-dispersive X-ray spectroscopy (EDS) analysis, as shown in Figure 3. Figure 3a shows the dark-field STEM image of the cross-sectional area of the Ti_3_C_2_T_*x*_/MoS_2_/sapphire structure, demonstrating parallel dense stacking between the 10-nm-thick Ti_3_C_2_T_*x*_ layer and the 2-nm-thick MoS_2_ layer, where the middle bright layer is MoS_2_ and the top and bottom adjacent layers are Ti_3_C_2_T_*x*_ and the sapphire substrate, respectively. Figure 3b–f shows elemental EDS mapping. Figure 3b,c shows the Ti and F EDS maps, indicating the composition of the top Ti_3_C_2_T_*x*_ layer. The S and Mo EDS maps in Figure 3d,e confirmed the middle bright MoS_2_ layer. The Al EDS map in Figure 3f shows the sapphire substrate. Figure 3g shows the cross-sectional STEM image at a higher magnification, confirming that Ti_3_C_2_T_*x*_ had nine layers and MoS_2_ had three layers. Using the intensity line scan in Figure 3h, plotted along the vertical line in Figure 3g, we observed that the vdW gap between Ti_3_C_2_T_*x*_ layers was ∼11 Å, which is consistent with the XRD result, and the vdW gap in the MoS_2_ layer was ∼6.4 Å, similar to previous reports.^41^ Importantly, the vdW gap between Ti_3_C_2_T_*x*_ and MoS_2_ was relatively small (∼6.7 Å), almost 3 times lower than that obtained via the SC method.^7^Figure 3i shows the mapping of the conductivity of the Ti_3_C_2_T_*x*_ thin film on the MoS_2_ surface, which was within the range of 7,100–12,000 S/cm, demonstrating the good conductivity and uniformity of the Ti_3_C_2_T_*x*_ thin film deposited via the MDC method (Figure S12).

**Figure 4:**
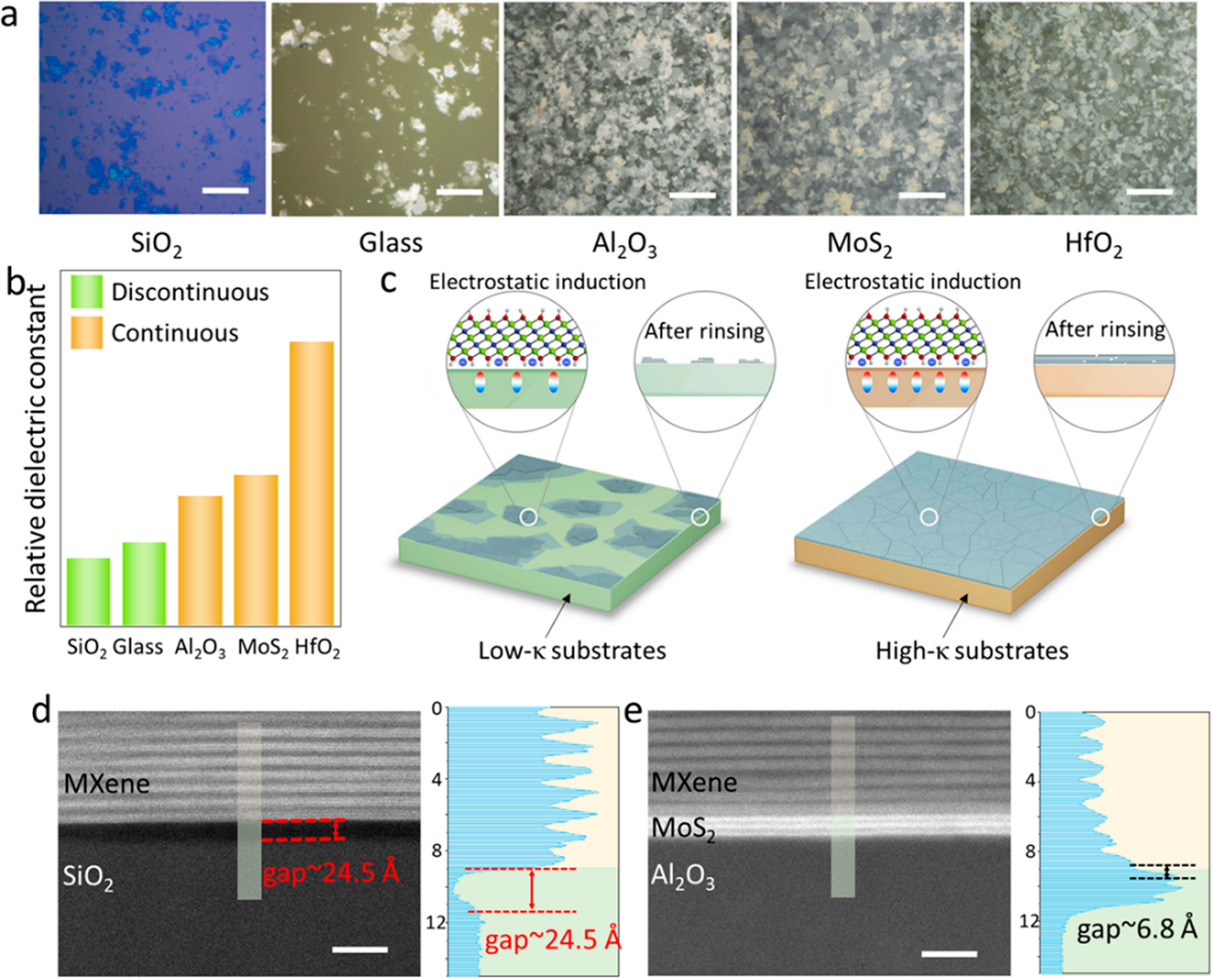
Substrate effect on the film quality using the MDC method. (a) Optical images of Ti_3_C_2_T_*x*_ films formed on different substrates. (b) Relative dielectric constant of different substrates. (c) Schematic of the Ti_3_C_2_T_*x*_ film formation mechanism (green, materials with low dielectric constants; yellow, materials with high dielectric constants). High-resolution cross-sectional STEM images and related intensity profiles of the line scans of (d) Ti_3_C_2_T_*x*_/SiO_2_ and (e) Ti_3_C_2_T_*x*_/MoS_2_/sapphire. (Scale bars: a, 30 μm; d,e, 5 nm.)

To understand the MXene film formation mechanism, we deposited Ti_3_C_2_T_*x*_ films using the MDC process onto different substrates, including SiO_2_, glass (Corning Alkaline Earth Boro-Aluminosilicate glass), Al_2_O_3_, MoS_2_, and HfO_2_, as shown in Figures 4a and S13. Using the optical microscope, we found that only isolated flakes were attached to SiO_2_ and glass, while uniform and continuous films with full coverage were deposited on the other substrates. Meanwhile, the Ti_3_C_2_T_*x*_ films fabricated on MoS_2_ and HfO_2_ show better flatness than that fabricated on Al_2_O_3_, as shown in Figure S14. These results demonstrate that the MDC method enables better film coverage on relatively high dielectric (κ) substrates (Al_2_O_3_: ∼7, MoS_2_: ∼8, HfO_2_: ∼16) compared to low-κ substrates (SiO_2_: ∼4, glass: ∼5), as shown in Figure 4b. Therefore, we hypothesize that the MXene film coverage is related to electrostatic interactions between the MXene film and substrate surface. Because Ti_3_C_2_T_*x*_ possesses a high density of negative surface charges, it introduces electrostatic polarization close to the surface of pristine substrates with positive charge terminations close to the MXene layer and negative charge terminations away from the MXene layer. These types of dipoles form mutual electrostatic attractions between multiple substrates and MXene layers. When using a low-κ substrate, the density of dipoles is smaller, resulting in the weak attraction and attachment of MXene to the substrate; therefore, isolated MXene flakes form on the low-κ substrates (Figure 4c). However, when using a high-κ substrate, a higher density of dipoles at the interface leads to stronger MXene/substrate interactions, thus resulting in the formation of the continuous MXene film on the high-κ substrate. To confirm the hypothesis, STEM was employed to visualize the interaction behavior between Ti_3_C_2_T_*x*_ and substrate, as shown in Figure 4d,e. Figure 4d shows the cross-sectional STEM image of Ti_3_C_2_T_*x*_ on (low-κ) SiO_2_ substrate. Using the line profile (right part of Figure 4d), we observed a large vdW gap of ∼24.5 Å between SiO_2_ and Ti_3_C_2_T_*x*_. However, the cross-sectional STEM image and corresponding line profile of (high-κ) MoS_2_/Ti_3_C_2_T_*x*_ sample demonstrate a considerably smaller vdW gap, ∼6.4 Å (Figure 4e). These images confirmed that low-κ substrates have a considerably weaker electrostatic attraction to the MXene layer, thereby increasing the rate of water penetration at the interface and causing film detachment. However, a small gap between the MXene layer and high-κ substrate can reduce the chance of water penetration and prevent the detachment of the MXene film.

**Figure 5:**
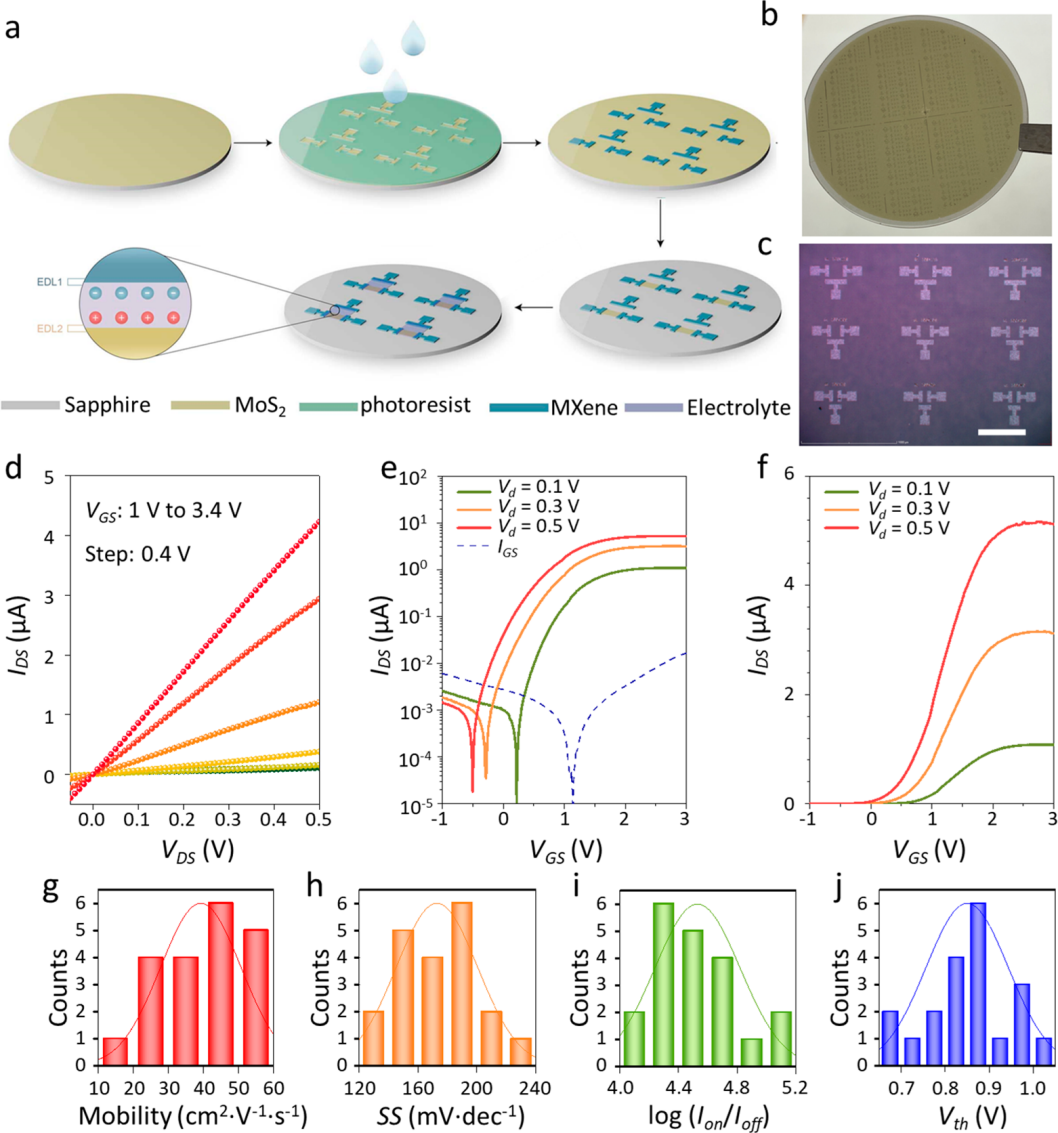
Performance of Ti_3_C_2_T_*x*_/MoS_2_ transistors. (a) Schematic of the Ti_3_C_2_T_*x*_/MoS_2_ transistor fabrication. (b) Optical image of Ti_3_C_2_T_*x*_ electrode arrays on a 2-in. MoS_2_ wafer. (c) Optical image of Ti_3_C_2_T_*x*_ electrode arrays on MoS_2_ (scale bar: 500 μm). (d) Output characteristics. Logarithmic-scale transfer curves (e) and linear-scale transfer curves (f) at different source/drain voltages. The dashed line in panel e represents the gate–source leakage current curve. Statistical distributions of effective mobility (g), *SS* (h), log(*I*_on_/*I*_off_) (i), and *V*_th_ (j) for 20 individual transistors from different points on the wafer.

The uniform Ti_3_C_2_T_*x*_ film prepared on the MoS_2_ surface was patterned using the standard cleanroom patterning process (Figure 5a). The photoresist was first patterned via conventional photolithography. The Ti_3_C_2_T_*x*_ film was then deposited on the patterned photoresist using the MDC method (the SC method failed to form a uniform Ti_3_C_2_T_*x*_ film on the patterned photoresist/MoS_2_ surface, as shown in Figure S15). After the uniform Ti_3_C_2_T_*x*_ thin film was formed on the substrate using the MDC method, the sample was immersed in acetone to remove the photoresist, and the uniform Ti_3_C_2_T_*x*_ pattern was achieved to form the source (S), drain (D), and side gate (G) electrodes on the MoS_2_ surface. Figure 5b shows the optical image of Ti_3_C_2_T_*x*_ electrode arrays on a 2-in. MoS_2_ wafer. The clean and uniformly patterned high-resolution Ti_3_C_2_T_*x*_ array is shown in Figures 5c and S16, and film detachment did not occur during the patterning process. Apparently, MXene nanosheets were tightly stacked, which ensures the effectiveness of electrode patterning. Furthermore, the MoS_2_ film was patterned in a rectangle as the semiconductor channel using conventional photolithography and dry etching. After spin-coating of the electrolyte on the MoS_2_ surface, the Ti_3_C_2_T_*x*_ MXene/MoS_2_ coplanar-gate transistor (with electrolyte dielectric) was finally obtained for the measurement of electrical transport properties.^42^

Figure 5d shows the output curve of a typical Ti_3_C_2_T_*x*_/MoS_2_ transistor device with a channel length of 150 μm and a channel width of 100 μm, demonstrating an obvious *n*-type characteristic. These *I*_DS_*–V*_DS_ output curves were obtained at different *V*_GS_ values in the range of 1–3.4 V with a step of 0.4 V. Figure 5e,f shows the transfer curves of the device obtained at different *V*_DS_ voltages (0.1, 0.3, and 0.5 V) on a logarithmic scale and a linear scale, respectively. The optimal device demonstrated a good *SS* value of ∼182.8 mV·dec^–1^, an acceptable *I*_on_/*I*_off_ of ∼1.01 × 10^5^, and a low threshold voltage (*V*_th_) of 0.85 V. Moreover, the maximum gate leakage current (*I*_GS_) was relatively low, ∼1 × 10^–8^ A, which is 100 times less than the effect of the source/drain on the current, indicating reliable device performance. To obtain the field-effect mobility of the MXene/MoS_2_ transistor device, the capacitance of the used electrolyte film was measured. The Nyquist plot was measured using a multichannel electrochemical workstation, as shown in Figure S17a. The frequency was scanned from 1 Hz to 100 kHz. As shown in Figure S17b, based on the equation *C* = (2π*fZ*″)^−1^, where *C* is capacitance, *f* is frequency, and *Z*″ is imaginary impedance, frequency-dependent specific capacitance (*C–f*) curves were measured. The specific capacitance at 1 Hz was 0.28 μF·cm^–2^.^43^ Hence, the field-effect mobility μ in the linear region was estimated to be ∼51.9 cm^2^·V^–1^·s^–1^ according to the calculated value. A comparison of the performance of devices with similar device structure (electrolyte-gated) is also summarized in Table S1, demonstrating the potential of the solution-processed MXene electrode. Moreover, we tested 20 devices with the same transistor size (Figure S18), and the devices exhibited a narrow distribution in performance, with a standard deviation of 39.3 ± 11 cm^2^·V^–1^·s^–1^ in mobility, 172.8 ± 27 mV·dec^–1^ in *SS*, 4.53 ± 0.28 in log(*I*_on_/*I*_off_), and 0.85 ± 0.09 V in *V*_th_, as shown in Figure 5g–j. These results demonstrate that the developed MDC method can produce large-area, uniform, high-quality, and damage-free MXene/MoS_2_ vdW arrays, which could enable more opportunities in 2D electronics.

## Conclusion

A modified MXene drop-casting process was developed for fabricating high-quality hydrophilic Ti_3_C_2_T_*x*_ thin films on hydrophobic MoS_2_ surfaces over large area. This solution process can be achieved at low temperatures (<100 °C) using DI water as the medium, which is simple, mild, and environmentally friendly. The obtained Ti_3_C_2_T_*x*_ films with a thickness of ∼10 nm demonstrated superior uniformity and continuity. Moreover, a highly flat Ti_3_C_2_T_*x*_/MoS_2_ vdW interface was reported with a gap of <1 nm. The MDC-processed Ti_3_C_2_T_*x*_ films can be reliably patterned using standard cleanroom photolithography and lift-off process owing to MXene-introduced MoS_2_ surface polarization phenomenon. Using the Ti_3_C_2_T_*x*_ pattern as the source, drain, and gate contact electrodes and MoS_2_ as the channel, we fabricated MXene–MoS_2_ transistors. The good contact between Ti_3_C_2_T_*x*_ and MoS_2_ allowed our Ti_3_C_2_T_*x*_–MoS_2_ transistor to achieve an average electron mobility of ∼40 cm^2^·V^–1^·s^–1^, on/off current ratios exceeding 10^4^, and subthreshold swings of under 200 mV·dec^–1^. This work enables the direct deposition of uniform hydrophilic MXene (Ti_3_C_2_T_*x*_) thin films on hydrophobic semiconductor (MoS_2_) surfaces with a high-quality MXene/MoS_2_ vdW interface. Furthermore, this MDC process can be applied to other transition metal dichalcogenides because they have properties (dielectric constants) similar to those of MoS_2_,^44^ enabling a wide range of applications in 2D nanoelectronics.

## Experimental Section

### Synthesis of Ti_3_C_2_T_*x*_

Ti_3_C_2_T_*x*_ MXene was prepared using a previously reported procedure.^7,45^ Note that 1 g of Ti_3_AlC_2_ MAX powder (400 mesh, purchased from Lanzhou Kai Kai Ceramic Materials Co. Ltd.) was slowly added to a mixture of 1 mL of HF (49%), 6 mL of HCl (12 M), and 3 mL of deionized (DI) water and stirred at 42 °C for 15 h. Subsequently, the mixture was extracted out and rinsed with DI water repeatedly until the pH value was 6. Then, 20 mL of aqueous LiCl solution (0.75 M) was added to the sediment and stirred for 20 min at room temperature. Next, four additional centrifuge cycles were performed to wash the Li^+^ intercalated multilayer MXene sediment. Finally, the MXene suspension was collected by centrifugation at 500 rpm for 10 min.

### Growth of the MoS_2_ Film

The MoS_2_ film was prepared on a sapphire substrate through the epitaxial phase conversion method previously reported by our group.^46^

### MXene/MoS_2_ Fabrication

Ti_3_C_2_T_*x*_ thin film/MoS_2_ was fabricated via a modified drop-casting (MDC) method. First, 0.15 mL of Ti_3_C_2_T_*x*_ MXene suspension (∼3 mg/mL) was dropped on the pristine MoS_2_/sapphire surface (1 × 1 cm^2^). Then, the Ti_3_C_2_T_*x*_ suspension/MoS_2_/sapphire was placed on a hot plate at 90 °C. After the Ti_3_C_2_T_*x*_ MXene suspension completely dried, we waited for another 10 min and then removed the substrate from the hot plate. When the substrate was cooled to room temperature, a drop of DI water was placed on the substrate surface to completely cover the thick Ti_3_C_2_T_*x*_ film. After 1 min, DI water was used to rinse the substrate surface, the thick Ti_3_C_2_T_*x*_ MXene film was peeled off, and the Ti_3_C_2_T_*x*_ thin film/MoS_2_ on the sapphire substrate was obtained.

### Patterned MXene/MoS_2_ Fabrication

The patterned Ti_3_C_2_T_*x*_ thin film/MoS_2_ was fabricated via the MDC method and a lift-off technique. First, the patterned photoresist (PR) on MoS_2_ was prepared through the conventional photolithography process. The AZ1512HS PR (Microchemicals) was spin-coated on the MoS_2_/sapphire substrate at 3000 rpm for 0.5 min and then baked at 100 °C for 1 min. Then, the mask aligner equipment (EVG6200∞) was employed to photopattern the PR. After UV exposure, the PR/MoS_2_/sapphire substrate was developed using the AZ 726 MIF developer (Microchemicals) for 20 s. After drying with nitrogen, the MDC method was used to fabricate the Ti_3_C_2_T_*x*_ thin film on the patterned PR/MoS_2_/sapphire substrate. Subsequently, the sample was immersed in acetone, the PR was removed under ultrasound treatment, and the patterned Ti_3_C_2_T_*x*_ thin film/MoS_2_/sapphire substrate was obtained.

### MXene/MoS_2_ Transistor Fabrication

The patterned Ti_3_C_2_T_*x*_ thin film electrodes on MoS_2_ were fabricated as mentioned above. To pattern the MoS_2_ film, the conventional photolithography process was used to pattern the PR on the MoS_2_ film; then, a plasma dry etching process was employed to remove the PR-uncovered area of the MoS_2_ film. After spin-coating the electrolyte on the substrate, the MXene/MoS_2_ transistor was obtained. Note that a ∼3 nm Al_2_O_3_ layer was grown on the channel surface to improve the contact between MoS_2_ and the electrolyte.

### Materials and Device Characterization

The surface morphologies of Ti_3_C_2_T_*x*_ thin films were visualized by optical microscopy (Zeiss AXIO Scope), scanning electron microscopy (SEM) (Nova Nano, FEI), and atomic force microscopy (AFM) (Bruker, Dimension Icon SPM). A Bruker D8 Advance XRD system with CuKα radiation (λ = 1.5406 Å) was employed to obtain the X-ray diffraction (XRD) patterns of Ti_3_C_2_T_*x*_ thin films. A focused ion beam (FIB) technique (Helios G4, FEI) was used to fabricate the transmission electron microscopy (TEM) sample. The TEM image was obtained using a Titan G2 60-300 (FEI), equipped with a spherical aberration corrector for an imaging system, at an acceleration voltage of 300 kV. Raman spectra were collected by a Wintec Apyron Raman spectrometer with a 633 nm laser source excitation. The hydrophilicity of MoS_2_ films was demonstrated by testing static contact angles using a contact angle system (OCA 35, DataPhysics, Filderstadt, Germany). A VMP3 multichannel electrochemical workstation (Bio-Logic) was used to measure the frequency-dependent capacitance and phase angle of the electrolyte. An Agilent B1500A semiconductor device analyzer equipped with a microprobe station (Summit-11600 AP, Cascade Microtech) was employed to determine the performances of transistors.

## Supporting Information Available

Note discussing the importance of substrate hydrophilicity in film deposition process; AFM image and statistical analysis of the thickness distribution of Ti_3_C_2_T_*x*_ MXene (Figure S1); zeta potential distribution of Ti_3_C_2_T_*x*_ MXene solution (Figure S2); optical images of MXene film dried at different temperatures (Figure S3); Raman spectra of Ti_3_C_2_T_*x*_ prepared at different temperatures and exposed to different Raman lasers (Figure S4); Ti 2p XPS spectrum of MXene-MDC-90 °C (Figure S5); cross-sectional structure of the deposited MXene film on MoS_2_/sapphire (Figure S6); SEM images and optical images of MXene films fabricated by different methods (Figures S7 and S8); contact angle of MoS_2_ treated with different times of UVO treatment (Figure S9); photos of photoresist/MoS_2_ and MoS_2_ before and after UVO treatment (Figure S10); AFM images of MXene films fabricated by different methods (Figure S11); distribution diagram of the conductivity of Ti_3_C_2_T_*x*_ MXene thin film on MoS_2_ surface (Figure S12); optical images of Ti_3_C_2_T_*x*_ MXene films on different substrates using MDC method (Figure S13); AFM images of MXene films on Al_2_O_3_ and HfO_2_ (Figure S14); optical images of Ti_3_C_2_T_*x*_ MXene films deposited on patterned PR/MoS_2_ before the lift-off process (Figure S15); optical images of patterned MXene electrode arrays on MoS_2_ using MDC method (Figure S16); Nyquist plot and capacitance and phase angle–frequency plots of the electrolyte film (Figure S17); transfer characteristics of 20 individual transistors (Figure S18); comparison of the performance of the transistor to prior state of the art (Table S1) (PDF)
