# Supplementary material for: Large-Area Metal–Semiconductor Heterojunctions Realized via MXene-Induced Two-Dimensional Surface Polarization
Source: ACS Nano. 2023 Apr 20;17(9):8324–32. doi: 10.1021/acsnano.2c12684 (PMC10173692; doi:10.1021/acsnano.2c12684)
Supplement: Supplementary file 1 — nn2c12684_si_001.pdf [file nn2c12684_si_001.pdf]

Large-Area Metal-Semiconductor Heterojunctions Realized via MXene-Induced Two-Dimensional Surface Polarization

*Tianchao Guo[1‡], Xiangming Xu[1‡], Chen Liu[2], Yizhou Wang[1], Yongjiu Lei[1], Bin Fang[1], Lin Shi[1], Hang Liu[1], Mrinal K. Hota[1], Hala A. Al-Jawhari[3], Xixiang Zhang[1], and Husam N. Alshareef[1]**

[1]Materials Science and Engineering, Physical Science and Engineering Division, King Abdullah University of Science and Technology (KAUST), Thuwal 23955-6900, Saudi Arabia

[2]Applied Physics, Physical Science and Engineering Division, King Abdullah University of Science and Technology (KAUST), Thuwal 23955-6900, Saudi Arabia

[3]Department of Physics, King Abdulaziz University, Jeddah, 21551 Saudi Arabia

*Corresponding Author

*E-mail: husam.alshareef@kaust.edu.sa

1. Supplementary Note: Discussion on the importance of substrate hydrophilicity in film deposition process.

The interactions between MXene solutions and substrates can be described by Young's equations, *i.e.* $\gamma_{sg} = \gamma_{sl} + \gamma_{lg}\cos\theta$, where γ_{sg}, γ_{sl}, and γ_{lg} represent the interfacial energy between a solid and a gas, a solid and a liquid, and a liquid and a gas, respectively, and θ is the contact angle. Moreover, Dupre's equation, *i.e.* $W_{sl} = \gamma_{sg}+ \gamma_{lg} - \gamma_{sl}$, can describe work of adhesion (W_{sl}). Obviously, a good wetting ($\theta\rightarrow0$) is necessary for the MXene's deposition.

2. Supplementary Figures and table

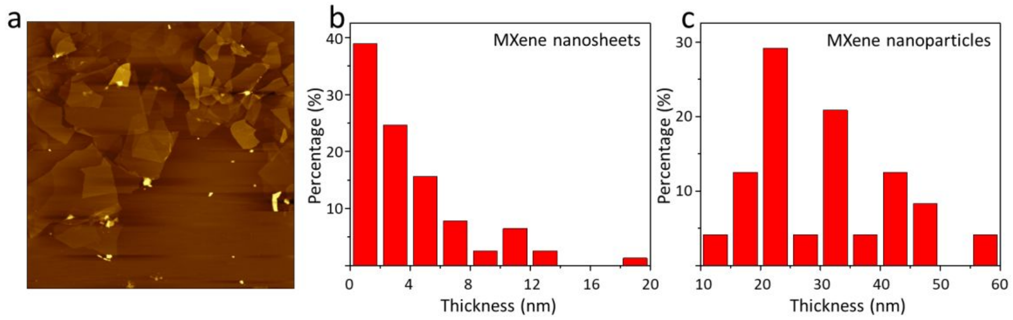

Figure S1. (a) The AFM image of $Ti_3C_2T_x$ MXene. Statistical analysis of the thickness distribution of $Ti_3C_2T_x$ MXene (b) nanosheets and (c) nanoparticles.

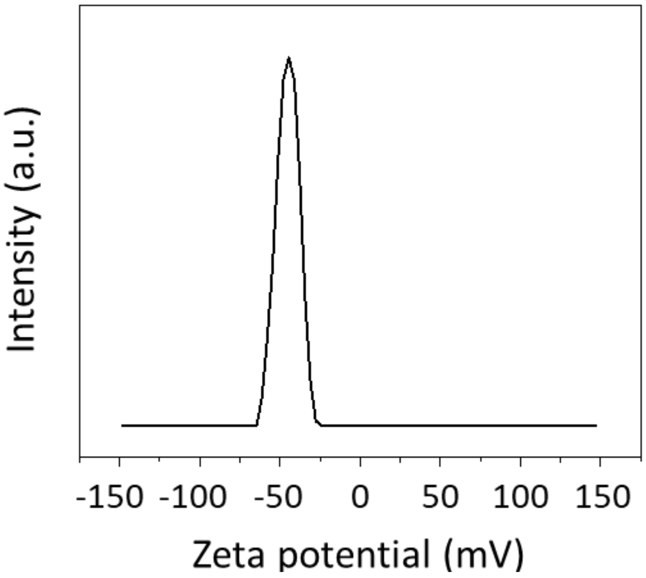

Figure S2. Zeta potential distribution of MXene solution.

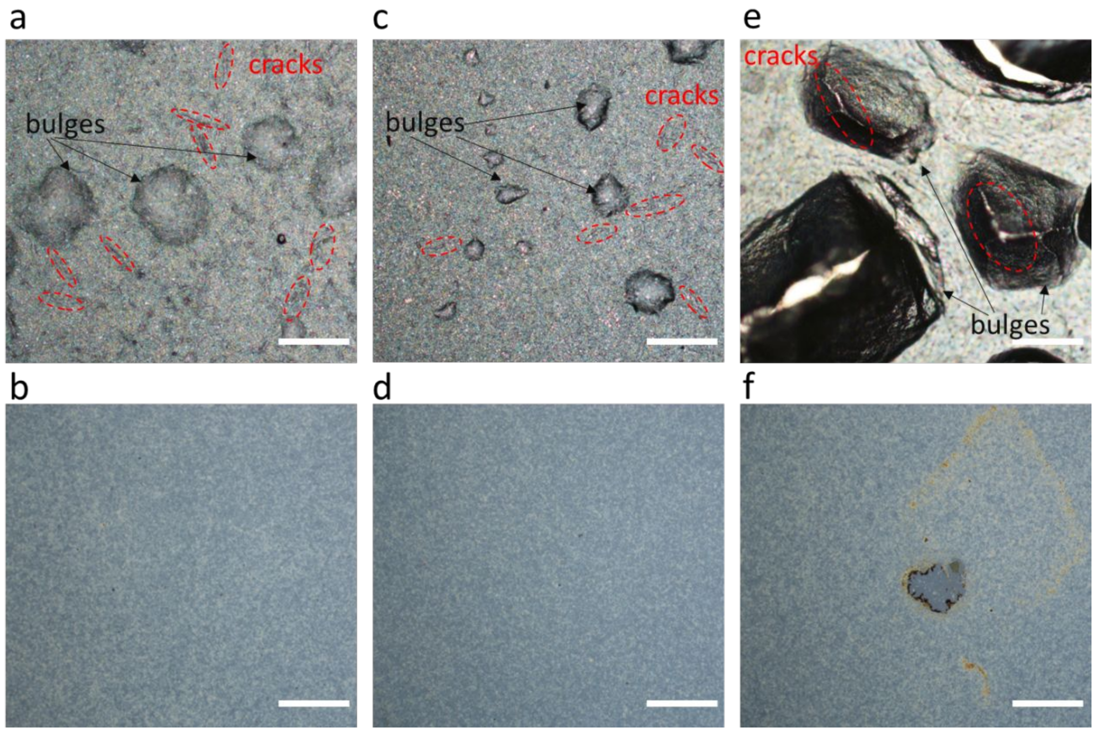

Figure S3. The optical images of MXene film dried at (a-b) 70 ℃, (c-d) 90 ℃, and (e-f) 110 ℃ before (Top) and after (Bottom) DI water rinsing. (Scale bar: 300 μm)

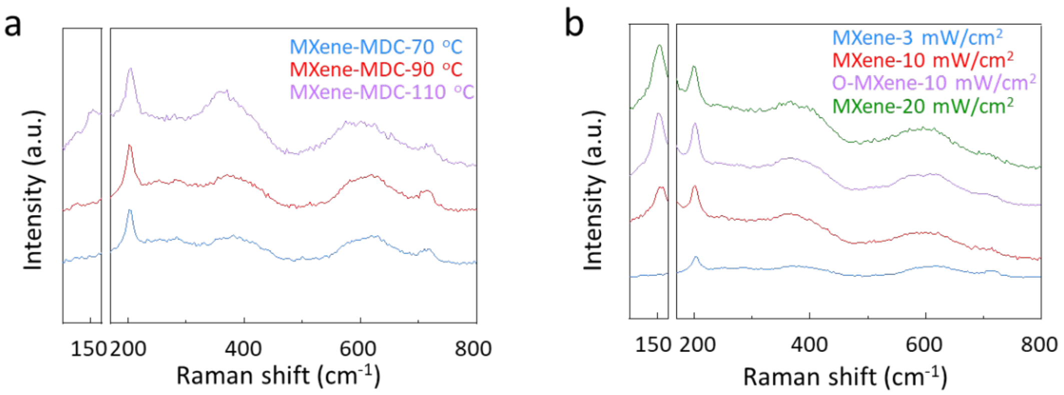

Figure S4. Characterization of $Ti_3C_2T_x$ MXene thin film on MoS_2 surface. Raman spectra of (a) $Ti_3C_2T_x$ MXene thin films prepared or processed at different temperature (MXene-MDC-70 ℃: 70 ℃; MXene-MDC-90 ℃: 90 ℃; MXene-MDC-110 ℃: 110 ℃) and (b) MXene-MDC-90 ℃ films after exposure to different Raman laser (MXene-3 mW/cm^2: 3 mW/cm^2; MXene-10 mW/cm^2: 10 mW/cm^2; MXene-20 mW/cm^2: 20 mW/cm^2; O-MXene-10 mW/cm^2: 10 mW/cm^2, after pre-irradiation for 2 mins at 10 mW/cm^2).

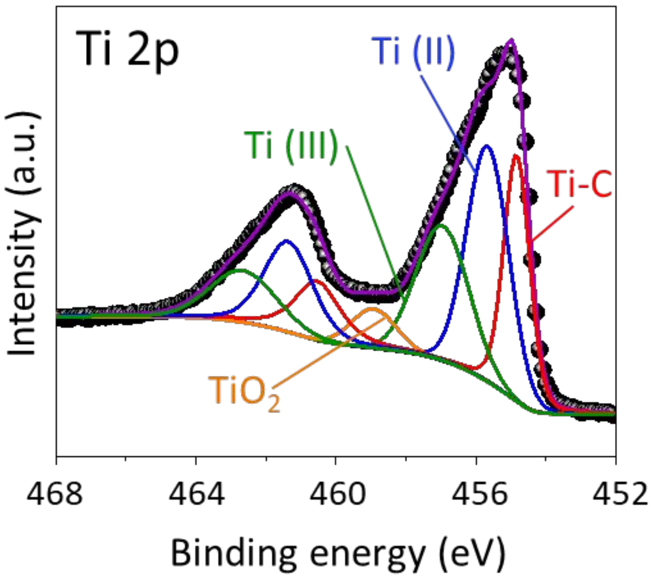

Figure S5. Ti 2p XPS spectrum of MXene-MDC-90 ℃.

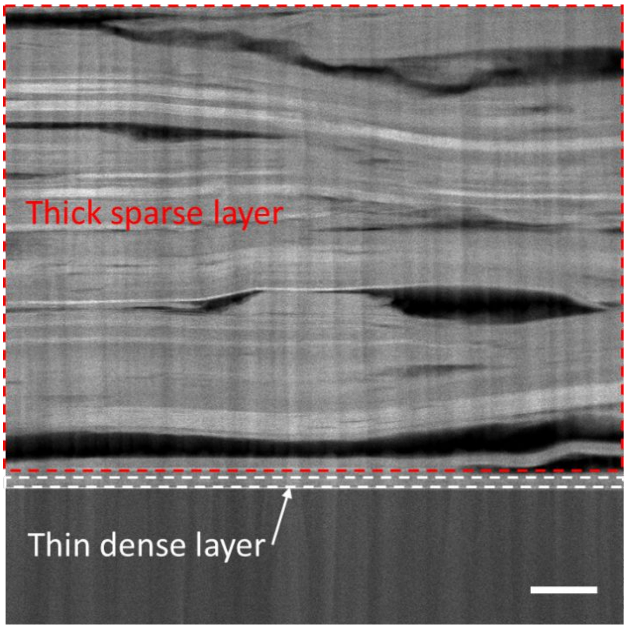

Figure S6. The cross-sectional structure of the deposited MXene film on MoS_2/sapphire. (Scale bar: 100 nm)

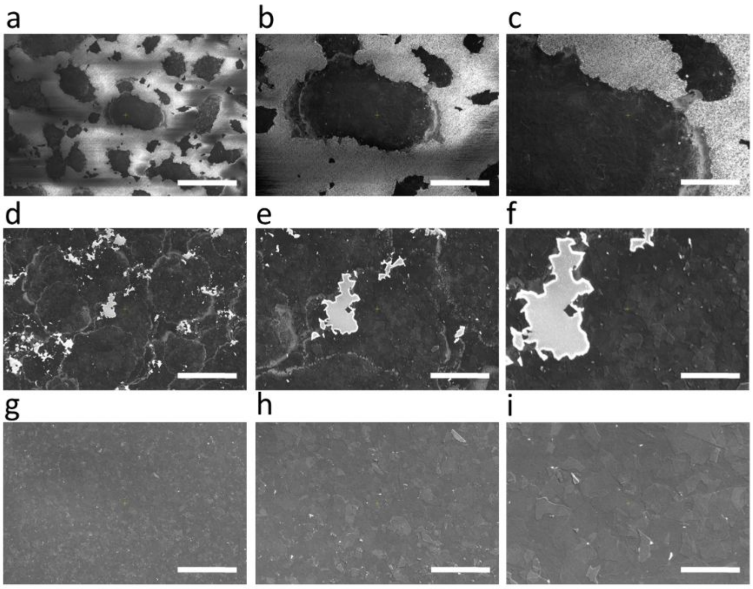

Figure S7. The SEM images of (a-c) SC-MXene/MoS_2, (d-f) SC-MXene/UVO-MoS_2, and (g-i) MDC-MXene/MoS_2. (Scale bar: a, d, and g, 300 μm; b, e, and h, 20 μm; c, f, and i, 10 μm;)

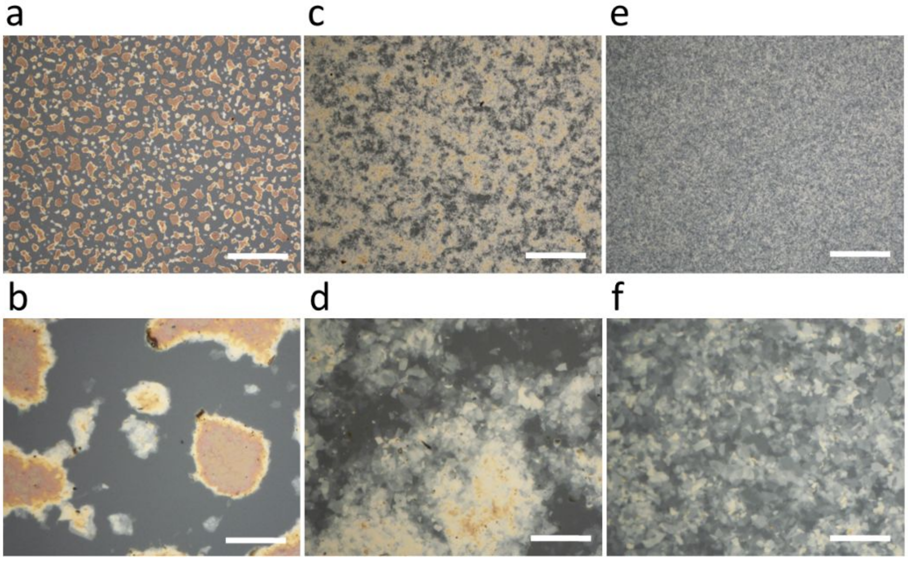

Figure S8. The optical images of (a-b) SC-MXene/MoS_2, (c-d) SC-MXene/UVO-MoS_2, and (e-f) MDC-MXene/MoS_2. (Scale bar: a, c, and e, 300 μm; b, d, and f, 30 μm)

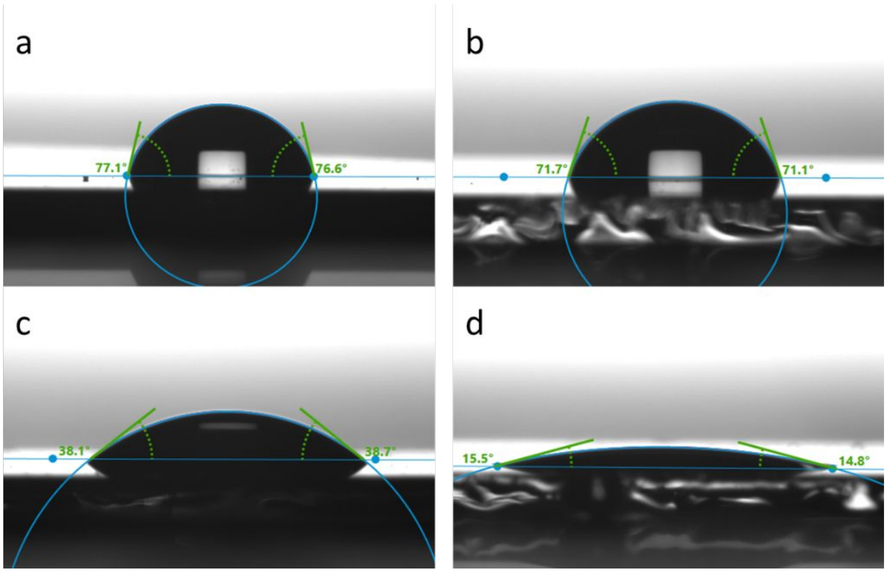

Figure S9. The contact angel of (a) pristine MoS_2, (b) UV Ozone 1 min treated-MoS_2, (c) UV Ozone 2 mins treated-MoS_2, and (d) UV Ozone 4 mins treated-MoS_2.

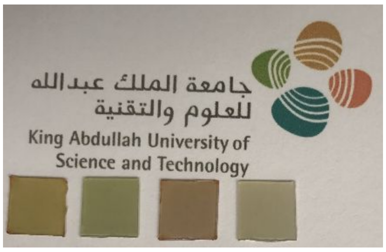

Figure S10. The optical images of PR/MoS_2, MoS_2, UV Ozone 4 mins treated-PR/MoS_2, UV Ozone 4 mins treated-MoS_2 (from left to right). (size: 1 cm × 1cm)

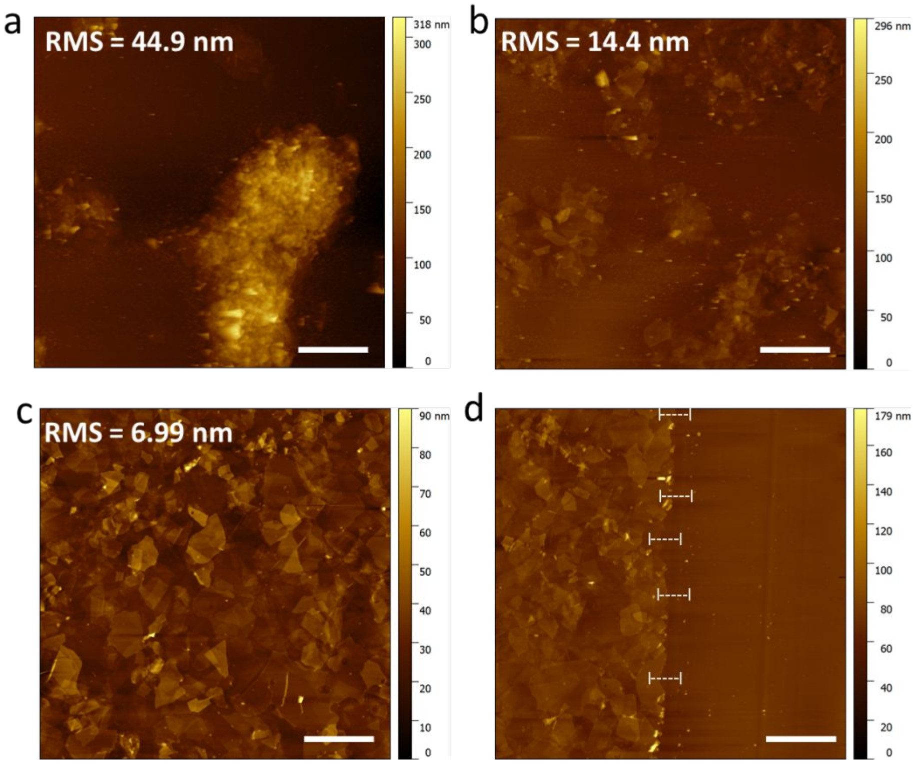

Figure S11. The AFM images of (a) SC-MXene/MoS_2, (b) SC-MXene/UVO-MoS_2, and (c-d) MDC-MXene/MoS_2. (Scale bar: 10 μm)

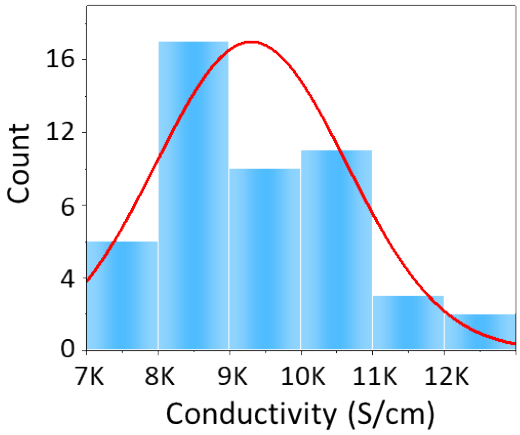

Figure S12. Distribution diagram of the conductivity of $Ti_3C_2T_x$ MXene thin film on MoS_2 surface.

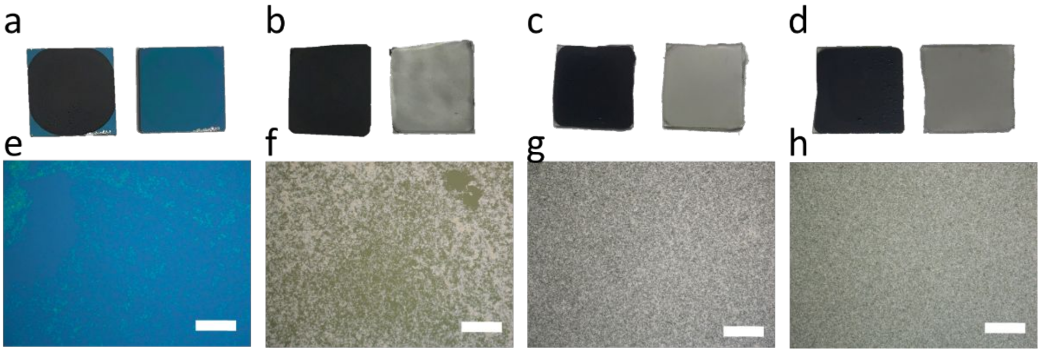

Figure S13. The optical images of $Ti_3C_2T_x$ MXene films/substrates (1 cm × 1cm) before (left) and after (right) water rinsing, (a) SiO_2, (b) glass, (c) Al_2O_3, and (d) HfO_2. (size: 1 cm × 1cm) The optical image of the $Ti_3C_2T_x$ MXene films formed on different substrates, (e) SiO_2, (f) glass, (g) Al_2O_3, and (h) HfO_2. (Scale bar: 300 μm)

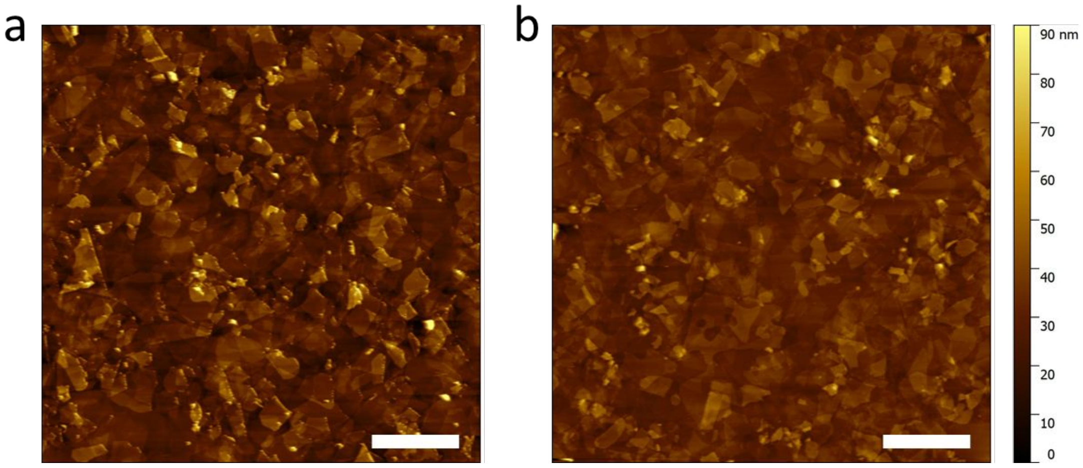

Figure S14. The AFM images of MXene film on (a) Al_2O_3 and (b) HfO_2. (Scale bar: 10 μm)

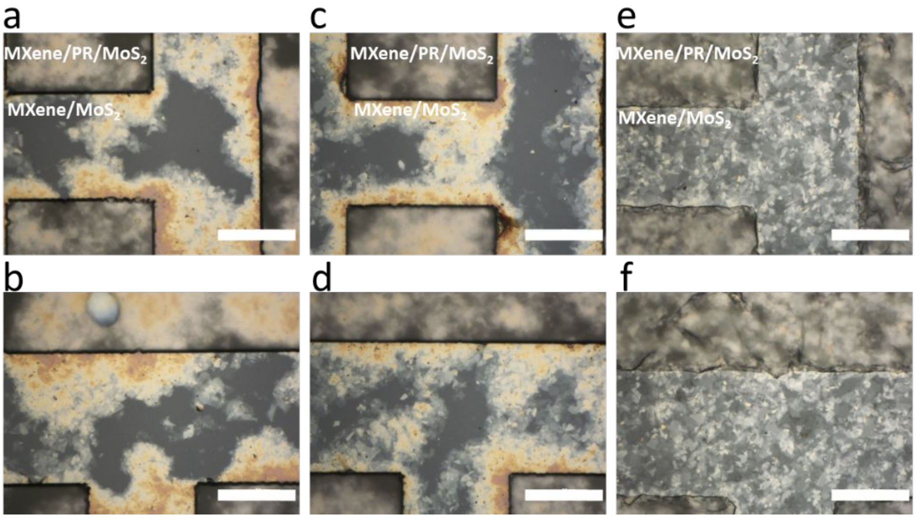

Figure S15. The optical images of $Ti_3C_2T_x$ MXene films deposited on patterned PR/MoS_2 before the lift-off process. (a-b) SC-MXene/MoS_2, (c-d) SC-MXene/UVO-MoS_2, (e-f) MDC-MXene/MoS_2. (Scale bar: 30 μm)

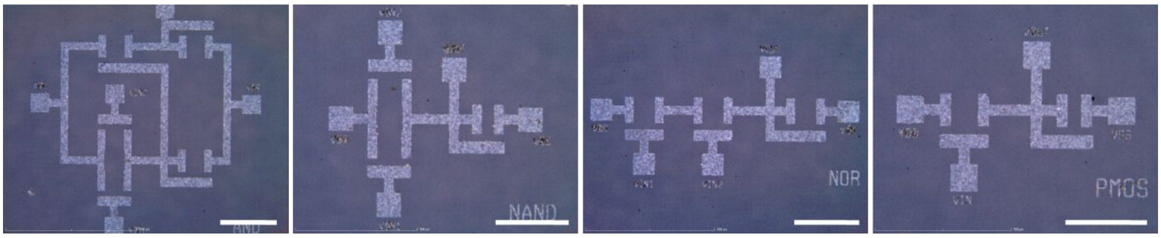

Figure S16. The optical images of patterned MXene electrode arrays on MoS_2 using MDC method. (Scale bar: 300 μm)

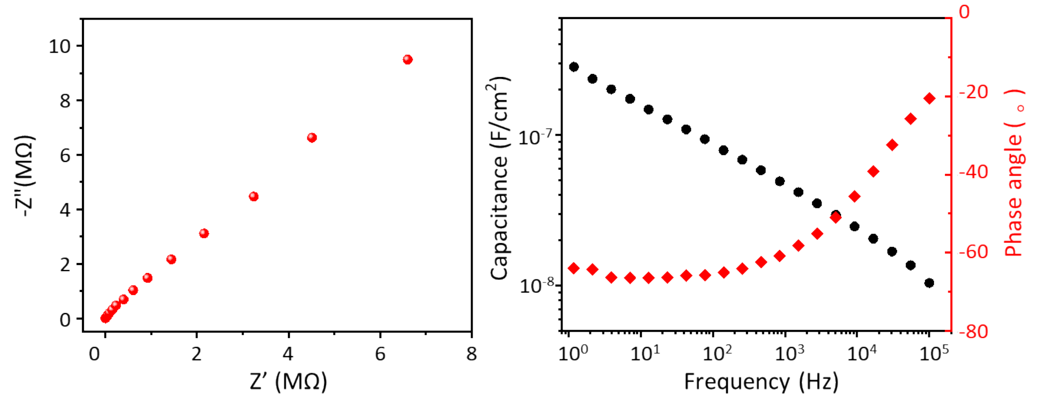

Figure S17. (a) Nyquist plot measured on the electrolyte film at room temperature. (b) The capacitance and phase angle-frequency plots of the electrolyte film.

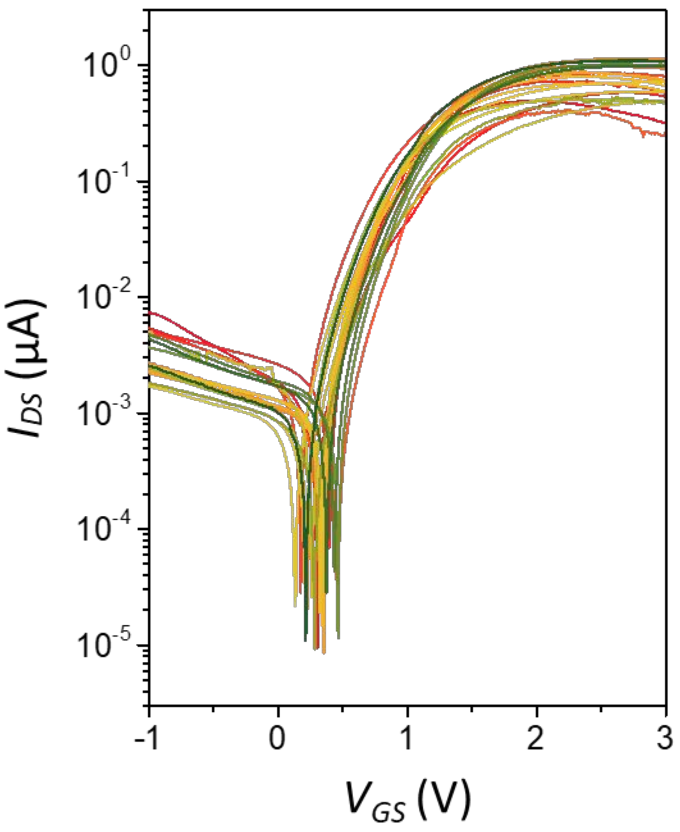

Figure S18. Transfer characteristics of 20 individual transistors (V_d = 0.1 V).

Table S1. Comparison of the performance to prior state of the art (with similar structure).

Channel	Electrode	Mobility ($cm^2 \cdot V^{-1} \cdot s^{-1}$)	SS ($mV \cdot dec^{-1}$)	I_{on}/I_{off}	Ref.
MoS_2	Au	10^{-2}	-	10^4	[1]
MoS_2	Au	14.3	110	10^4	[2]
MoS_2	Ti/Au	10	-	10^5	[3]
MoS_2	Ti/Au	2.04	50	10^5	[4]
MoS_2	Cr/Au	131	67	$>10^5$	[5]
MoS_2	Ni/Au	13	130	$>10^5$	[6]
WS_2	-	50	-	-	[7]
WSe_2	Pd	30	200	10^7	[8]
WSe_2	Ni/Au	55	70	$>10^5$	[6]
MoS_2	MXene	51.9	182.8	10^5	This work

REFERENCES

1. Stefano Ippolito; Adam G. Kelly; Rafael Furlan de Oliveira; Marc-Antoine Stoeckel; Daniel Iglesias; Ahin Roy; Clive Downing; Zan Bian; Lucia Lombardi; Yarjan Abdul Samad; Valeria Nicolosi; Andrea C. Ferrari; Jonathan N. Coleman; Samorì, P. Covalently interconnected transition metal dichalcogenide networks via defect engineering for high-performance electronic devices. *Nature Nanotechnology* 2021, 16, 592-598.

2. Hongwei Tang; Wei Niu; Fuyou Liao; Haima Zhang; Hu Xu; Jianan Deng; Jing Chen; Zhijun Qiu; Jing Wan; Yong Pu; Bao, W. Realizing Wafer-Scale and Low-Voltage Operation MoS_2 Transistors via Electrolyte Gating. *Adv. Electron. Mater.* 2020, 6, 1900838.

3. Jihun Mun; Hyeji Park; Jaeseo Park; DaeHwa Joung; Seoung-Ki Lee; Juyoung Leem; Jae-Min Myoung; Jonghoo Park; Soo-Hwan Jeong; Won Chegal; SungWoo Nam; Kang*, S.-W. High-Mobility MoS_2 Directly Grown on Polymer Substrate with Kinetics-Controlled Metal–Organic Chemical Vapor Deposition. *ACS Appl. Electron. Mater.* 2019, 1, 608-616.

4. Binmin Wu; Xudong Wang; Hongwei Tang; Wei Jiang; Yan Chen; Zhen Wang; Zhuangzhuang Cui; Tie Lin; Hong Shen; Weida Hu; Xiangjian Meng; Wenzhong Bao; Jianlu Wang; Chu, J. Multifunctional MoS_2 Transistors with Electrolyte Gel Gating. *Small* 2020, 16, 2000420.

5. Yongsuk Choi; Hyunwoo Kim; Jeehye Yang; Seung Won Shin; Soong Ho Um; Sungjoo Lee; Moon

Sung Kang; Cho, J. H. Proton-Conductor-Gated MoS_2 Transistors with Room Temperature Electron Mobility of >100 cm^2 V^{-1} s^{-1}. *Chem. Mater.* 2018, 30, 4527-4535.

6. Jiang Pu; Kazuma Funahashi; Chang-Hsiao Chen; Ming-Yang Li; Lain-Jong Li; Takenobu, T. Highly Flexible and High-Performance Complementary Inverters of Large-Area Transition Metal Dichalcogenide Monolayers. *Adv. Mater.* 2016, 28, 4111-4119.

7. Jinhuan Wang; Xiaozhi Xu; Ting Cheng; Lehua Gu; Ruixi Qiao; Zhihua Liang; Dongdong Ding; Hao Hong; Peiming Zheng; Zhibin Zhang; Zhihong Zhang; Shuai Zhang; Guoliang Cui; Chao Chang; Chen Huang; Jiajie Qi; Jing Liang; Can Liu; Yonggang Zuo; Guodong Xue; Xinjie Fang; Jinpeng Tian; Muhong Wu; Yi Guo; Zhixin Yao; Qingze Jiao; Lei Liu; Peng Gao; Qunyang Li; Rong Yang; Guangyu Zhang; Zhilie Tang; Dapeng Yu; Enge Wang; Jianming Lu; Yun Zhao; Shiwei Wu; Feng Ding; Liu, K. Dual-coupling-guided epitaxial growth of wafer-scale single-crystal WS_2 monolayer on vicinal a-plane sapphire. *Nature Nanotechnology* 2022, 17, 33-38.

8. Yu-Chuan Lin; Bhakti Jariwala; Brian M. Bersch; Ke Xu; Yifan Nie; Baoming Wang; Sarah M. Eichfeld; Xiaotian Zhang; Tanushree H. Choudhury; Yi Pan; Rafik Addou; Christopher M. Smyth; Jun Li; Kehao Zhang; M. Aman Haque; Stefan Fölsch; Randall M. Feenstra; Robert M. Wallace; Kyeongjae Cho; Susan K. Fullerton-Shirey; Joan M. Redwing; Robinson, J. A. Realizing Large-Scale, Electronic-Grade Two-Dimensional Semiconductors. *ACS Nano* 2018, 12, 965-975.
